# Supplementary material for: Enabling remote quantum emission in 2D semiconductors via porous metallic networks
Source: Nat Commun. 2020 Jan 7;11:5. doi: 10.1038/s41467-019-13857-0 (PMC6946668; doi:10.1038/s41467-019-13857-0)
Supplement: Supplementary file 1 — Supplementary Information [file 41467_2019_13857_MOESM1_ESM.docx]

**Enabling remote quantum emission in 2D semiconductors via porous metallic networks**

**­**

**Fonseca et al.**

**Supplemental Information**

**Supplementary Figures and Supplementary Discussion**


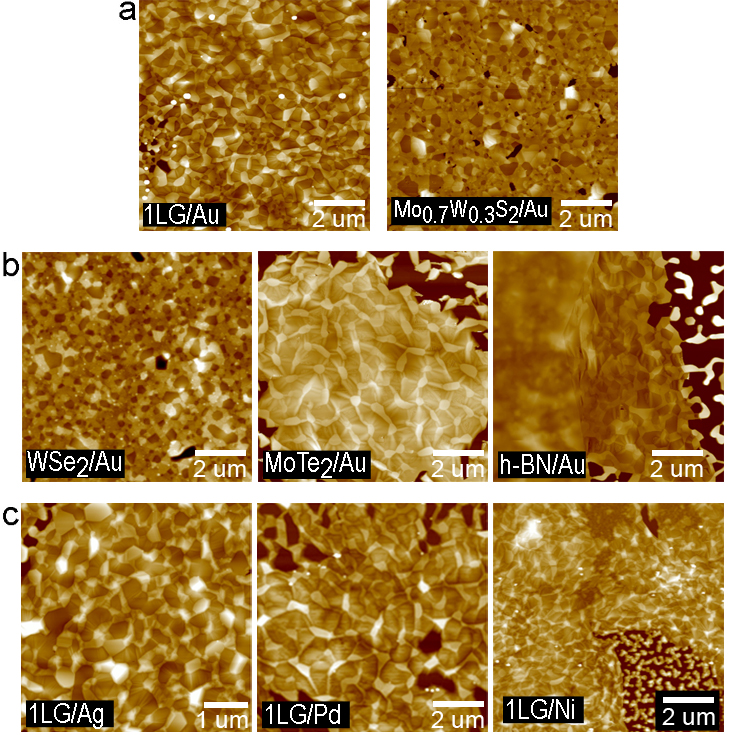


**Supplementary Figure 1.** AFM height scans of de-wetted metals capped with 2D crystals. (a) Left: CVD-grown graphene on Au annealed at 300 °C for 1 hr; right: MOCVD-grown Mo_0.7_W_0.3_S_2_ on Au annealed at 300 °C for 2 hr (note: MOCVD materials used in this study were provided by The Pennsylvania State University 2D Crystal Consortium − Materials Innovation Platform (2DCC-MIP)). (b) Mechanically-exfoliated crystals on Au. Left: WSe_2_ on Au annealed at 300 °C for 30 min; center: MoTe_2_ on Au annealed at 300 °C for 30 min; right: h-BN on Au annealed at 300 °C for 1.5 hr. (c) Other metals capped with CVD-grown graphene. Left: Ag annealed at 250 °C for 1 hr; center: Pd annealed at 500 °C for 1 hr; right: Ni annealed at 400 °C for 1 hr.


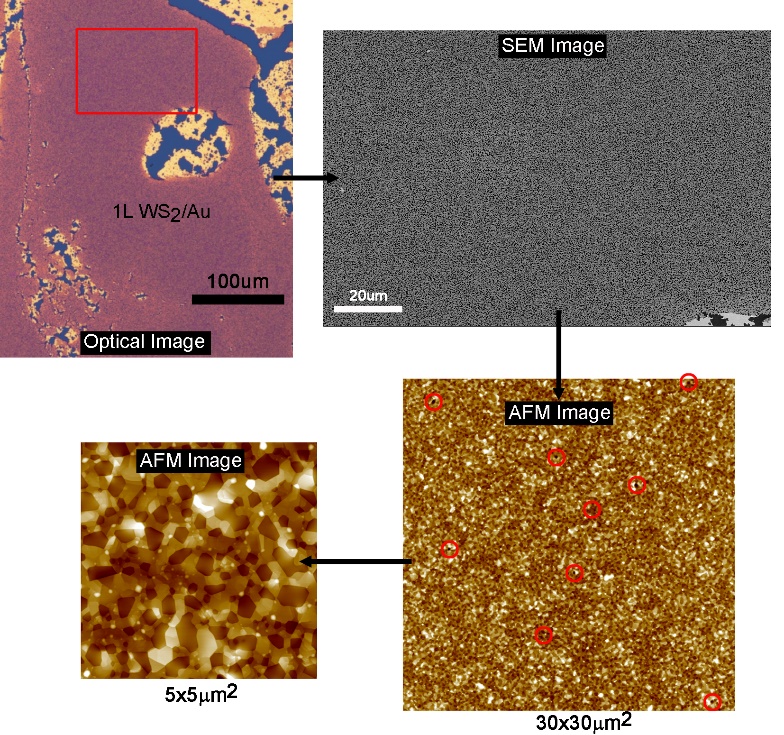


**Supplementary Figure 2.** Example showing the formation of tens-of-thousands of pores in an OPEN-WS_2_ films. From the 30x30um^2^ AFM image, we can identify nine (9) WS_2_ membranes which coat the inside of the Au pore (red circles).


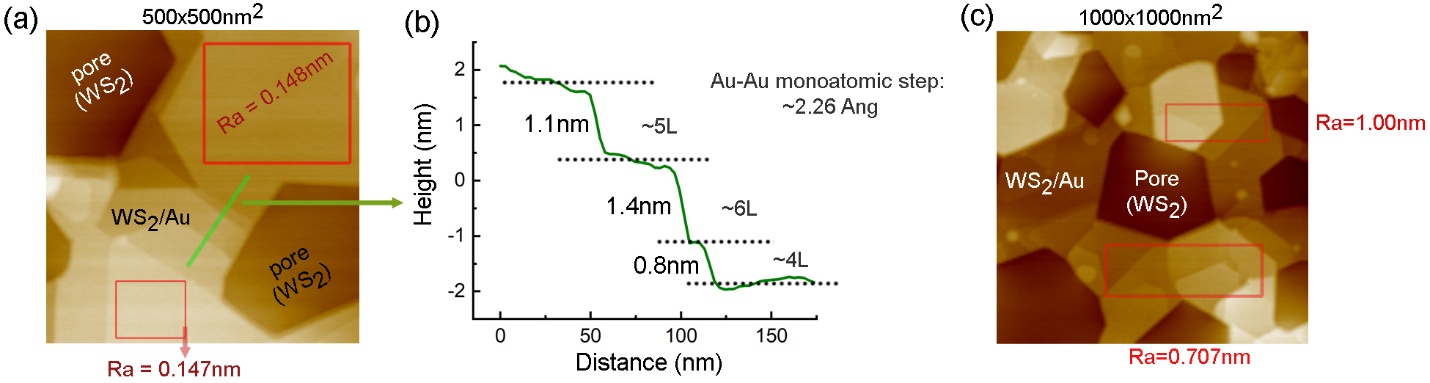


**Supplementary Figure 3.** Higher resolution AFM images of a WS_2_/Au sample annealed at 300°C. The surface roughness (R_a_) is analyzed for different regions (red boxes), ranging from individual Au terraces (a) to multi-terrace areas (c). (b) Example of height variation across the surface due to step bunching of monoatomic Au layers.


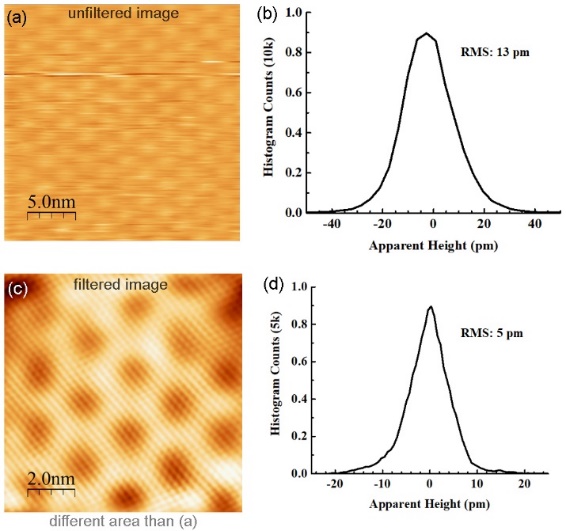


**Supplementary Figure 4.** STM images were taken in a ScientaOmicron LT STM under UHV (~10^-11^ Torr) conditions with LN_2_ cryostat cooling to 78K. Constant-current feedback parameters: +1.5 V tip bias, 100 pA setpoint in (a) and -0.5 V, 500 pA in (c). Electrochemically etched W tips were used, which were gold plated on sputter-cleaned Au(111) on mica. The sample was aligned under the STM tip by an optical window in the thermal shielding and long-range *ex situ* microscope. The resulting image data was analyzed with WSxM software for assignment of RMS roughness and is plotted in panels (b) and (d), corresponding to images (a) and (c), respectively. In both (a) and (c) the moiré pattern formed between MoS_2_/Au is clearly observed, indicating an atomically clean interface. Filtration for (c) consisted of a low-pass inverse-FFT filter.


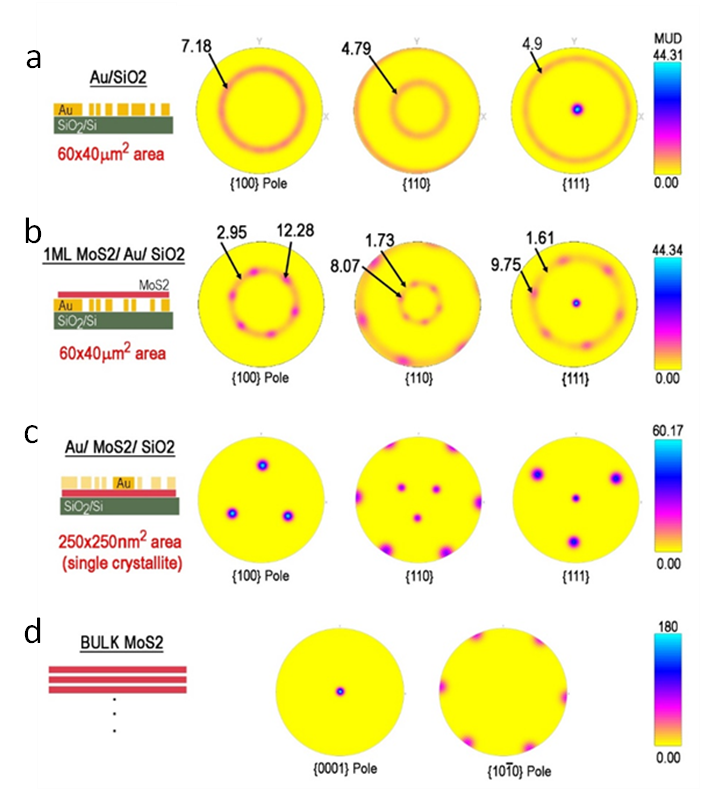


**Supplementary Figure 5.** Complete EBSD Pole plot series from Figure 1g in main text. Schemes *(left)* illustrate the analyzed sample structure and indicate the sampling area for the pole plots to their right. (a – b) Average pole plots from (a) uncapped and (b) MoS_2_-capped de-wetted gold films. (a) Au crystallites show (111) texture with random in-plane rotation. (b) Au crystallites show (111) texture with two main in-plane rotations offset by 60° (crystal rotation schemes shown in Supplementary Figure 6). (c) Average pole plots from a single Au crystallite de-wetted on a MoS_2_ monolayer, showing (111) texture and its 3-fold symmetry. (d) Average pole plots from a bulk MoS_2_ single-crystal flake.


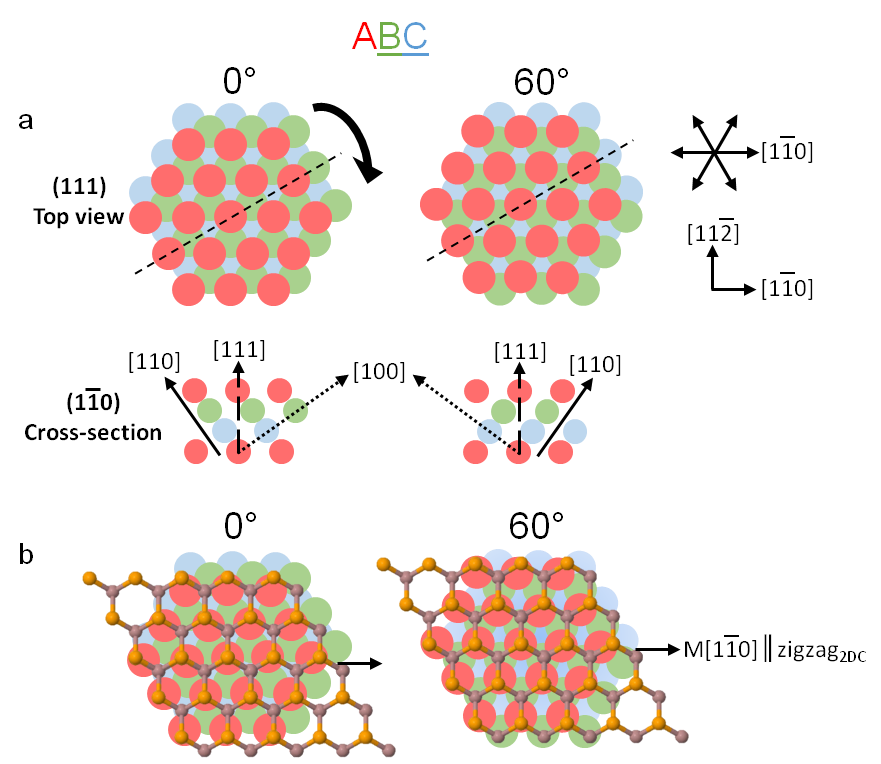


**Supplementary Figure 6.** Recrystallized gold orientation. (a) Top *(top)* and side *(bottom)* view of Au(111) planes stacks illustrating the surface degeneracy after a 60° rotation. Side view shows cross-sections at the ($1\bar{1}0$) planes indicated by the dashed lines above. (b) Illustration of the two main crystal alignment rotations (0° and 60°) of recrystallized gold to a 2DC layer on top. Black arrows indicate both the Au <$1\bar{1}0$> and the 2DC zigzag directions.


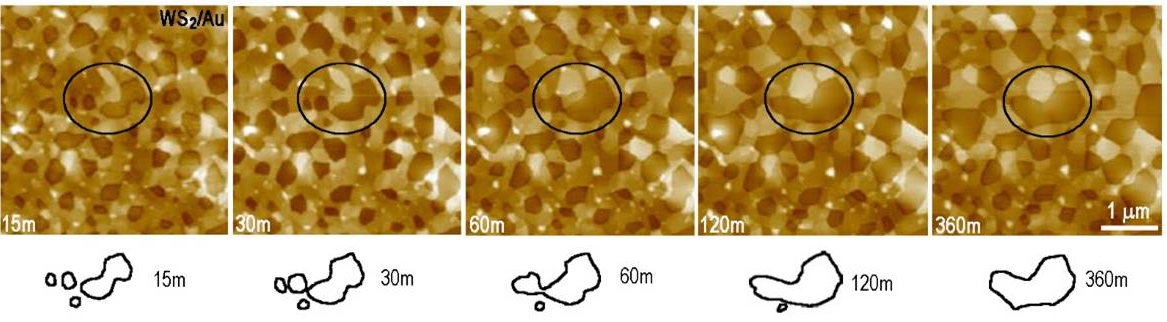


**Supplementary Figure 7.** Complete series of AFM height scan images in Fig. 2a, corresponding to every pore outline in Fig. 2b (image size = 4x4μm^2^).


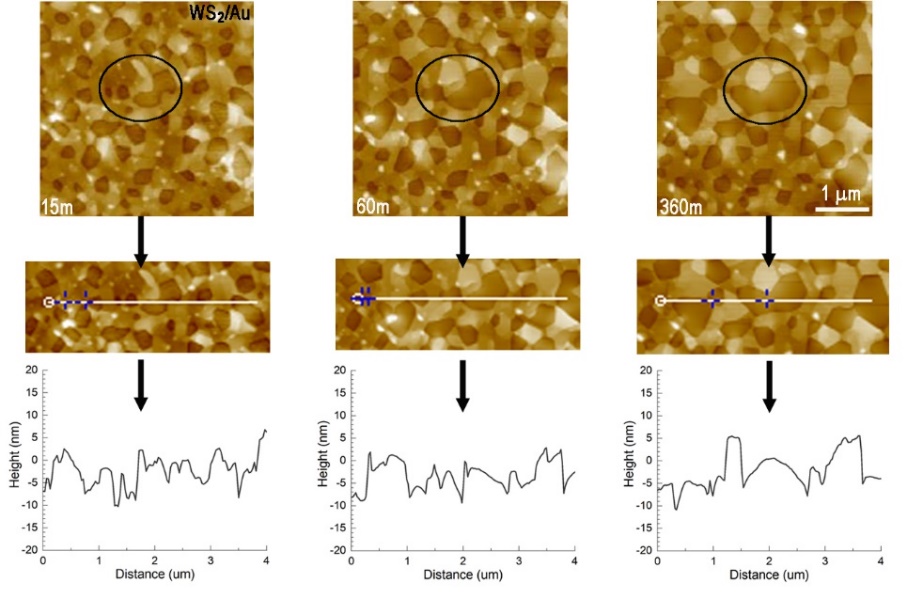


**Supplementary Figure 8.** AFM cross sections taken from Figure 2a of the main text.

Regarding sample roughness in the images shown in Figure 2a, the total image roughness factor (R_a_) increases with anneal time by ~0.6 nm. The measured image roughness (R_a_) for 15 minutes: R_a_=3.63 nm; 60min: R_a_=3.96 nm; and 360min: R_a_=4.20 nm. We note that this R_a_ analysis also includes height variations caused by the slightly depressed WS_2_ membranes, in addition to height variations in the Au layer. As a result, these ‘image R_a_’ values are higher than those from the crystalline Au film itself. As shown in Supplementary Figure 3 and 4, the individual Au terraces can be atomically flat with step bunching occurring across the surface as metal re-distributes to form pores.


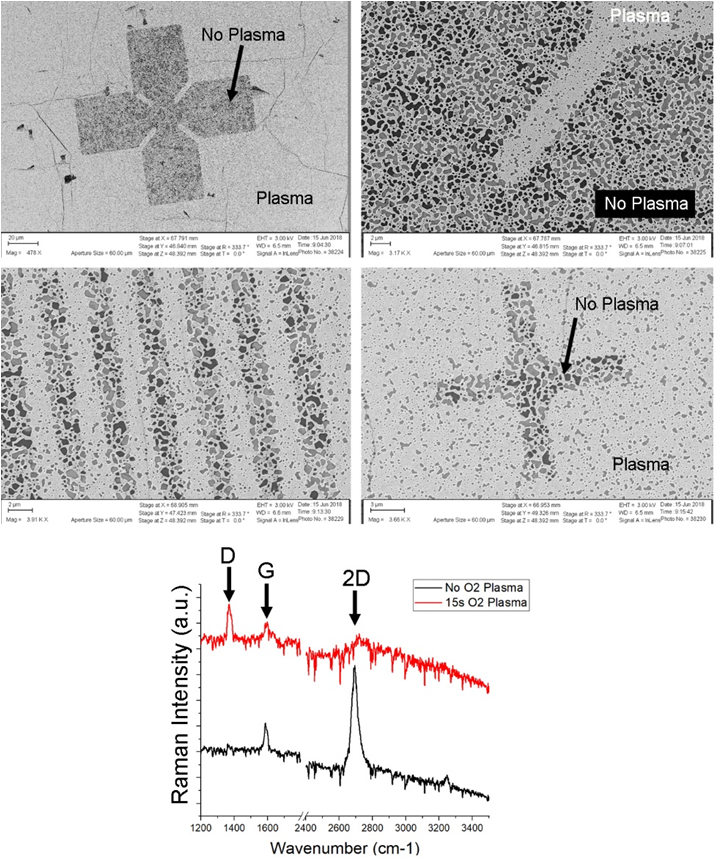


**Supplementary Figure 9.** SEM images showing different examples of patterned de-wetting. The CVD graphene/Au sample was patterned with photoresist, then exposed to O2-plasma for 15sec (Plasma preen tool). The photoresist was removed in acetone and the sample was annealed at 300C for 1 hour in flowing Ar/H_2_. The smallest patterned features examined here were 2 μm bars (bottom left SEM image). The Au film beneath graphene exposed to plasma de-wets at a slower rate compared to ‘pristine’ graphene regions. The lowest panel shows Raman spectra taken from a 15s O_2_-plasma region and a pristine graphene region. The Raman signal has additional noise due to the Au underlayer.


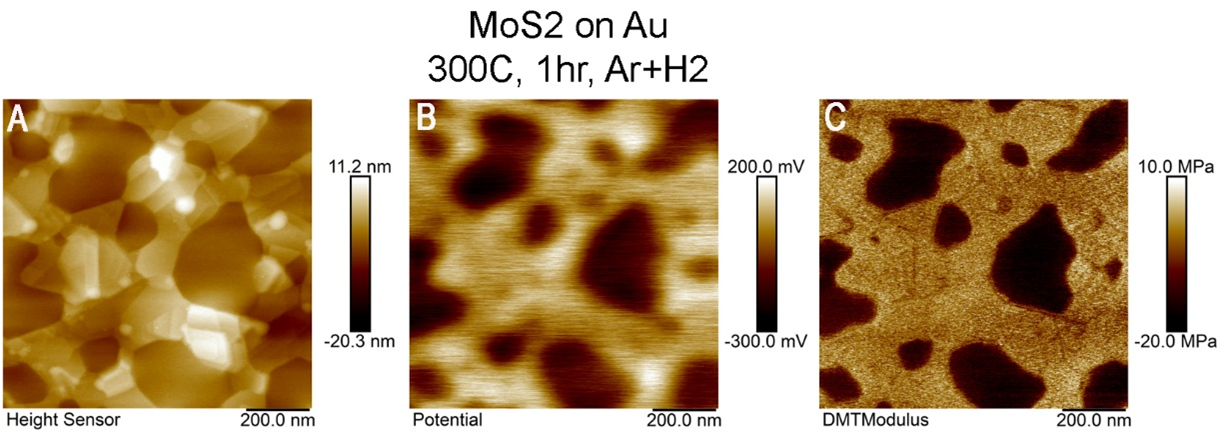


**Supplementary Figure 10.** AFM image showing an example of an exfoliated MoS_2_ film on Au/SiO_2_ after annealing at 300°C for 1hr. (A) Height, (B) Surface potential, and (C) DMT Modulus images were acquired during the same scan. The surface potential scan in (B) is shown in Figure 2d of main text. In the DMT Modulus scan, the suspended MoS_2_ membranes appear to have a (relative) reduced modulus as compared to the Au-supported MoS_2_ regions.


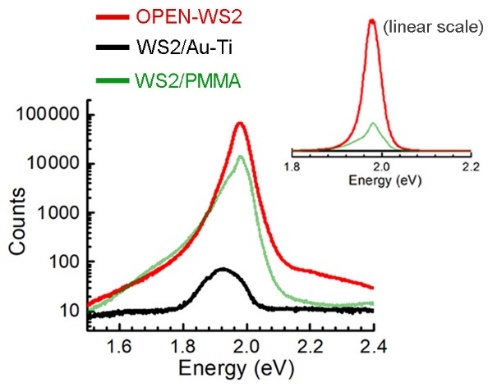


**Supplementary Figure 11.** Comparison of room temperature PL of WS_2_ from Figure 3c, including a PMMA substrate.


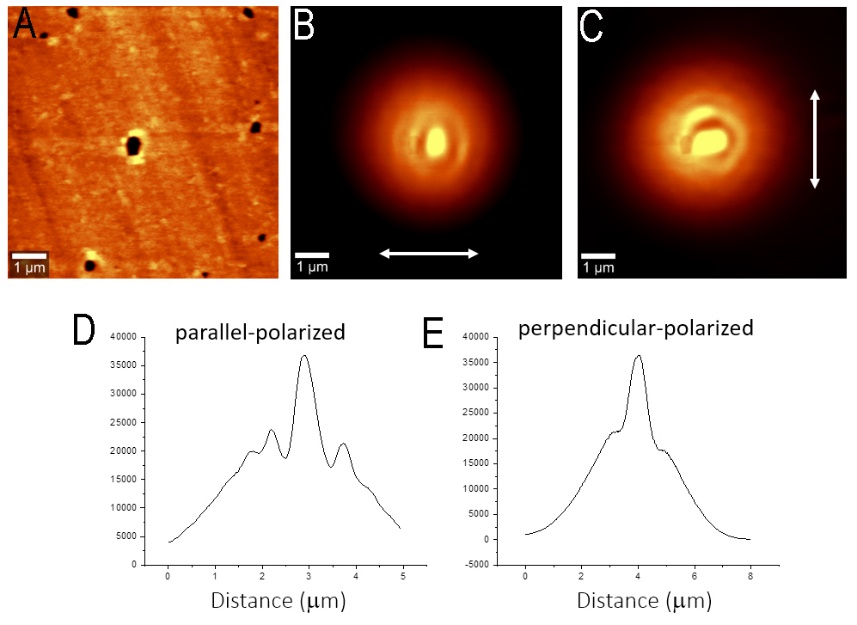


**Supplementary Figure 12.** (A) AFM of an isolated pore in an OPEN-WS_2_ film. (B,C) SNOM measurements under illumination polarization direction indicated by white arrow. (D,E) Intensity cross-section from the SNOM image in ‘B’. Section taken parallel to the illumination polarization direction (D) shows oscillations while the section taken perpendicular (E) shows no oscillations.

SPPs can transmit their energy across gaps, where the transmission probability depends on the gap distance. In gold films, the transmission rate across a 1 μm gap is ~50%, for a 200 nm gap is ~80%, and for ≤30 nm gaps it approaches 100% [^1^]. For comparison to our system, we note that the OPEN-Au films do not have linear ‘gaps’ as studied by Flynn et al. [^1^], but instead have a continuous conducting surface of gold. From this, it is reasonable to assume the transmission values in [^1^] serve as a LOWER bound for comparing transmission losses of pores to that of true metal gaps.

In Figure 2 of the manuscript we show an example of the pore distributions with annealing a WS_2_/Au sample. In this experiment, we measured the ensemble average pore areas of 0.028μm^2^ (resulting in 8.9% pore area coverage) and 0.154μm^2^ (resulting in 29.6% pore area coverage-- discussed in main text), following annealing treatments of 15 minutes and 360 minutes, respectively. Assuming a simple circular hole shape (area= π*r^2^ =1/4*π*d^2^), the ensemble average pore diameters in these samples would be approximately 190nm and 440nm, respectively. For similar ‘gap’ dimensions from Flynn et al. [^1^], we could estimate a LOWER bound for SPP transmission efficiencies at 60-80% across a wide range of OPEN film morphologies.

Figure 4 was acquired using a ‘sandwich’ sample with TWO layers of gold− Au/WSe_2_/Au − as illustrated in Figure 1a (bottom) in the main text (also see Supplementary Figure 12(A) below). The average pore diameter in this region is 230 nm ±88nm (Supplementary Figure 12(B)), which would correspond to a gap transmission efficiency from Flynn et al. [^1^] of approximately 80%. In addition, from the pore areal density we can calculate an average linear pore density (L_d_) of ~1.1 pores/μm. Taken together, to estimate loss associated with linear scattering between points ‘A’ and ‘B’ due to the pores, we assume that the intensity drops by a fixed percentage determined by an average pore transmission rate:

$I(x)=I_{0}\left( T^{x*L_{D}} \right)$ (1)

Applying our experimental L_d_ and a variable transmission efficiency (T), we compare the loss associated with pore scattering and that associated with the known exponential Ohmic losses in the metal itself (i.e., I= I_o_e^-x/γ^ ; note: In the linear approximation, we do not apply the 2D-radial spreading term (1/x)). As shown in Supplementary Figure 12(C) below, a pore transmission rate of T=50% would produce similar loss to that expected from Ohmic loss in gold at λ=532nm excitation.

To estimate the total loss due to an Ohmic component and a pore transmission component we use the product of:

$I(x)=I_{0}\left( e^{\frac{-x}{}} \right)\left( T^{x*L_{D}} \right)$ (2)

In Supplementary Figure 12(D), we plot the normalized data from the most intense PL in the “727nm” plasmon region shown in Figure 4f of the main text. A good overlap of Supplementary Equation 2 occurs with a pore transmission rate of approximately 94%. As reasonably expected, the best transmission rate for the porous metal structure is larger than that measured for a perfect 230nm gap assessed by Flynn et al. [^1^], where we estimated the pore transmission here to be about 18% larger as compared to the true gap transmission in [^1^]. Finally, we reemphasize that this assessment characterizes the most efficient combined process for exciton excitation, SPP excitation/propagation, and exciton re-excitation. From the plot in Figure 4f of the main text, intensity points that fall below these most intense PL data represent less efficient exciton/SPP propagation/exciton pathways in the OPEN film.


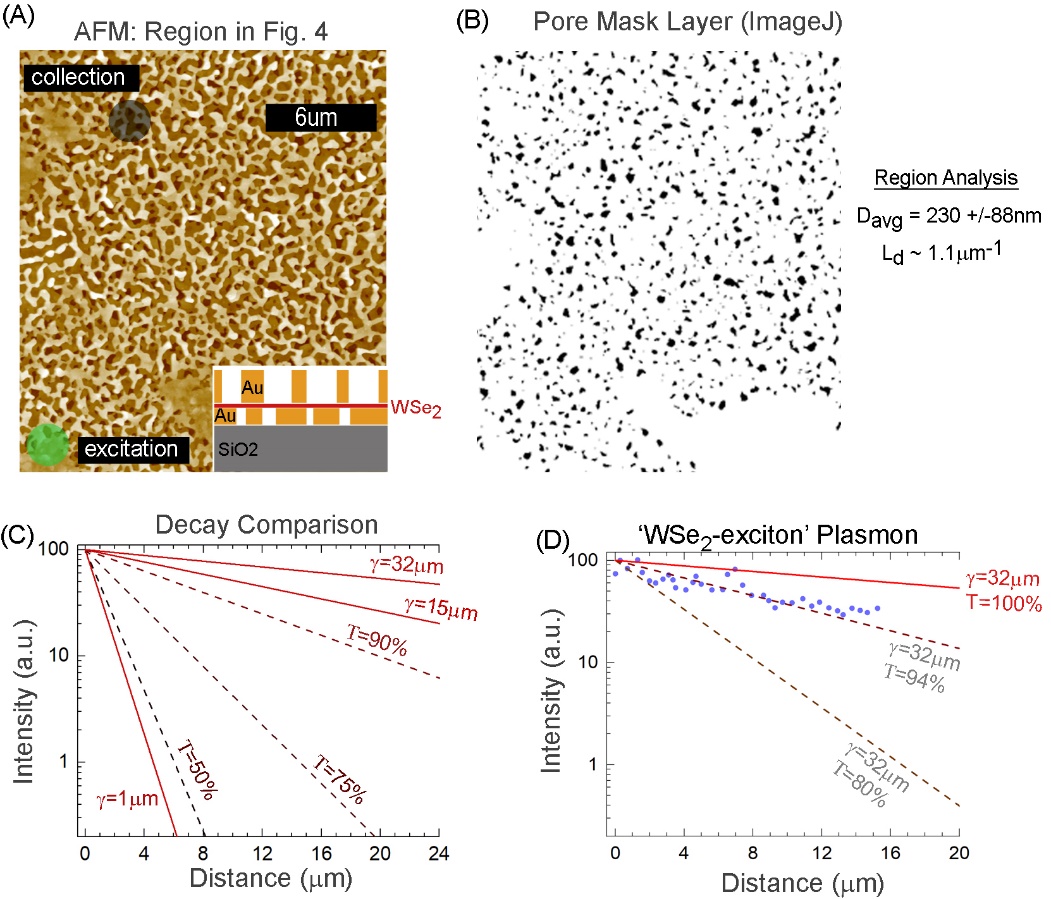


**Supplementary Figure 13.** (A) AFM from the region in Figure 4 of the main text showing positions of the central collection spot, and the position of the 17μm excitation spot. (B) Particle counting via ImageJ software to produce a mask of the pores with exposed WSe_2_. (C) Plot comparing loss due exponential decay (I=I_o*_e^-x/γ^) and that for fixed transmission loss from Supplementary Equation 1. (D) Plot showing normalized data (peak intensity) from the 727nm plasmon region, together with Supplementary Equation 2 using different transmission values.


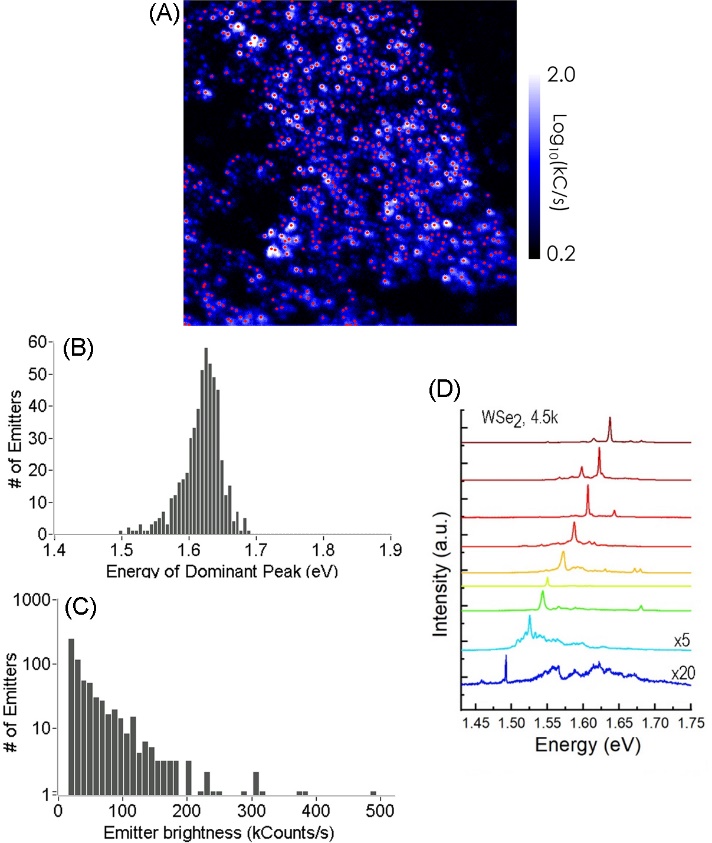


**Supplementary Figure 14.** Optical characterization from a wider region of the sample described in Figure 4 of the main text. (A) Red dots identify sites of narrow emitters. (B, C) Histograms showing emitter peak energy and brighteness, respectively. (D) Individual emitters selected from (A).

We have performed COMSOL simulations to examine how well the in-plane and out-of-plane dipoles couple into SPP modes of the metal, similar to Zhou Y. et al. [^2^]. These simulations show that the in-plane dipoles couple into SPP modes much more strongly when a pore is present in the metal, as compared to a continuous metal film as studied in [^2^]. The resulting |E|^2^ intensity is close to three to four orders-of-magnitude more intense in the presence of a pore (Supplementary Figure 14.1 and 14.2).


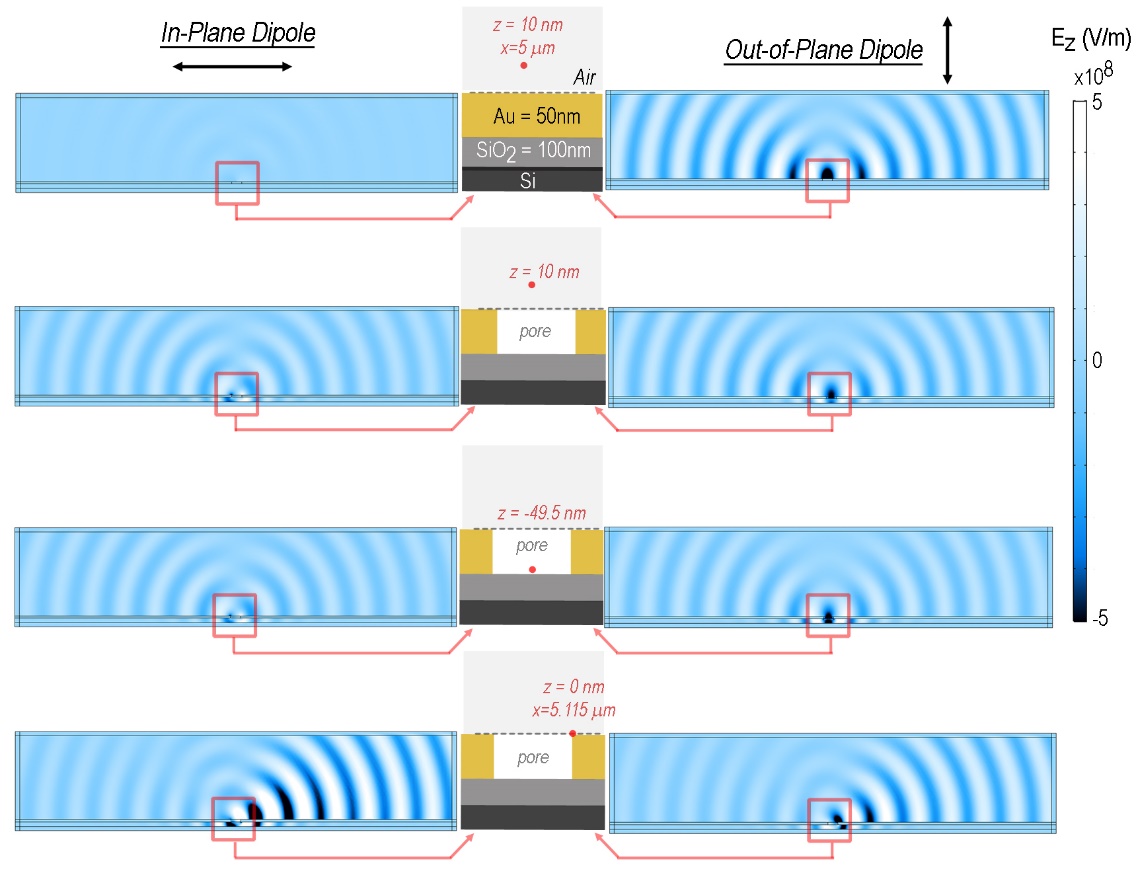


**Supplementary Figure 15.** 2D COMSOL simulations showing the field intensity component E_z_ from an oscillating dipole (red dot in schematic), both in-plane and out-of-plane, above a gold surface with and without a pore. The dipole position is labeled relative to the dotted line at the Au surface and is either 10 nm above the Au surface (z=10 nm) or at the bottom of the pore (z= -49.5 nm) just above the SiO_2_ surface. The simulation length is 10 μm with the dipole centered at x=5 μm. The material thicknesses are all the same as labeled in the top schematic (note: schematic not to scale). The pore diameter is 230 nm.


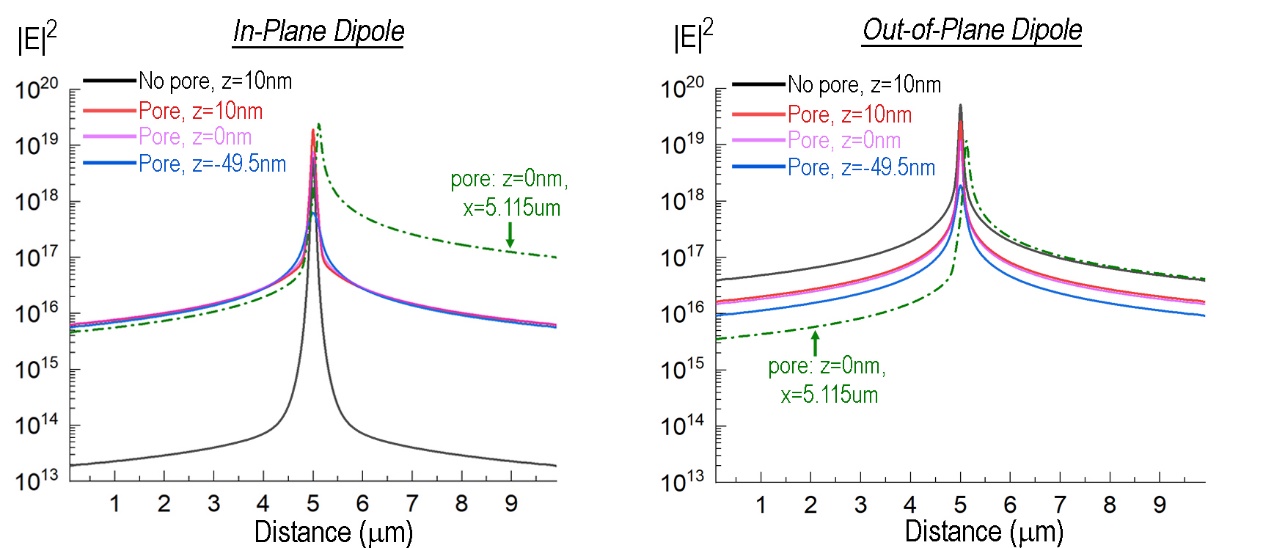


**Supplementary Figure 16.** Intensity cross-section taken from the 2D COMSOL simulations in Supplementary Figure 14.1 at 50nm above the surface, where the dipole is centered at x=5 μm (pore diameter=230nm). The plot shows the square of the field intensity (|E|^2^). The presence of the pore results in significant improvement for the in-plane dipole coupling, where |E|^2^ is close to three orders-of-magnitude larger than a continuous film (left plot) away from the emitter site. When the dipole is offset to the edge of the pore (x=5.115um), there is an additional order-of-magnitude increase in the |E|^2^ toward the side closest to the dipole.

To help visualize how SPP energy interacts with an individual pore site, we examine cross-sectional profiles from an individual pore in Supplementary Figure 19. We selected a pore that is 10.5 μm from the launching site to verify that a measurable amount of SPP energy is still available to interact with a dipole located within the pore. While the SPP power within the continuous metal film is negligible, SPP intensity enters within the pore and there are intensity ‘hot spots’ both at the top surface (Au/Air) and at the bottom surface (Au/SiO2) and thereby can interact with a 2D semiconductor that is either suspended above or coating the inside of pore.


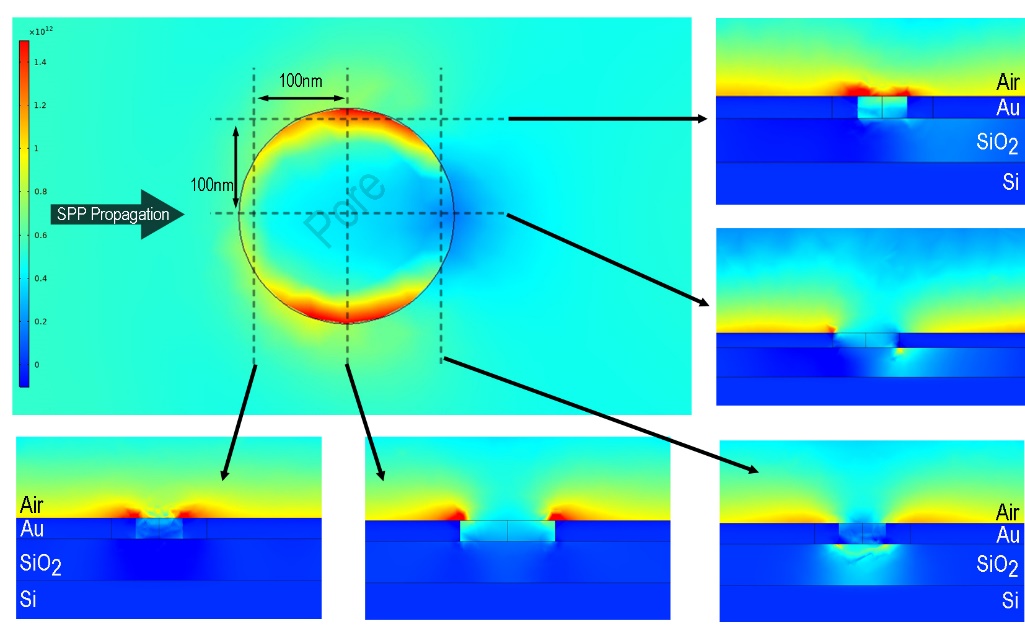


**Supplementary Figure 17.** 3D COMSOL simulation showing the 727 nm time averaged x-component of the power propagation across an individual pore (diameter = 230nm, Au thickness =50nm, SiO2 thickness = 100nm). This data is taken from the tenth pore shown in Supplementary Figure 19 and is 10.5μm from the launch site.

We have performed an additional control experiment at the same region mapped in Figure 4 to examine the extent of scattered 532nm light from the OPEN film structure. In this control, we perform the same measurement as shown in Figure 4b, but use a 532 nm band-pass filter to only collect scattered 532 nm light (and not PL light). The collected 532nm signal is attenuated (via neutral density filter) so as to match the maximum count rate of the PL measurement (red data points). In Supplementary Figure 16 below, we observe that the intensity of scattered 532 nm light drops off at a greater rate than the PL intensity starting at approximately 0.5 μm away from the excitation spot. By 3 μm from the excitation spot, the signal intensity is at the baseline intensity of ~200counts/s.


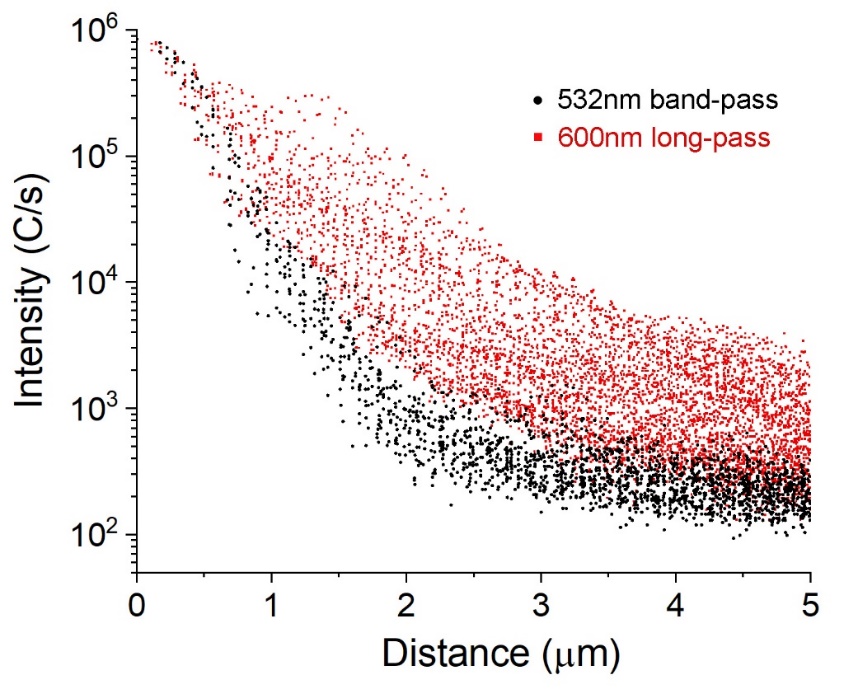


**Supplementary Figure 18.** Plot of light intensity (counts/s) versus distance acquired at the same location as in Figure 4b of the main text. The 600nm long-pass filter data is that shown in Figure 4f, and the control experiment (532nm band-pass) is included for comparison.

SPPs are excited by two different wavelengths in our sample: (i) via the direct laser field through in-coupling at pore sites, and (ii) via excitons excited in the 2D semiconductor. As such, the total PL decay should be described by

$I=I_{b}+{I_{532nm}\frac{1}{x}e^{\frac{-x}{{}_{532nm}}}+I}_{727nm}\frac{1}{x}e^{\frac{-x}{{}_{727nm}}}$(3)

where λ refers to the exciting wavelength, I_λ_ is the initial wavelength specific intensity at the launching site, γ_λ_ is the wavelength specific decay length, and I_b_ is the background signal. The additional 1/x term arises from SPPs radiating in all directions in the 2D plane. To gain insight into the most efficient transmission pathways in the OPEN film, we apply Supplementary Equation 3 to the most intense PL data in Figure 4f. The most intense PL points represent the highest efficiencies for energy in-coupling, SPP propagation, and energy out-coupling.


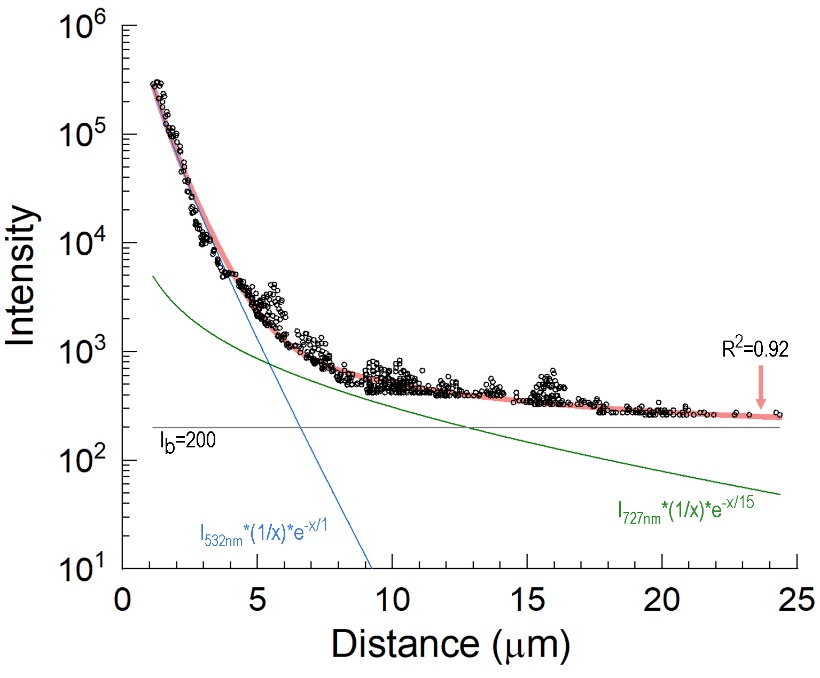


**Supplementary Figure 19.** Plot of the most intense PL data (peak intensity) taken from the remote PL map in Figure 4b, together with a fit from Supplementary Equation 3 (red line). The individual components of the fit are labeled on the plot, where I_b_=200, I_532nm_ = 9.6E5, I_727nm_ = 6E3, γ_532nm_ = 1, γ_727nm_ = 15.

Confined SPPs lose energy as they propagate due to Ohmic losses. The propagation length as a result of Ohmic losses alone is given by $L={(2Im\left[ \beta\right])}^{-1}$ where $\beta={\lambda_{i}}/{2\pi\sqrt{{\varepsilon_{m}}/{{(\varepsilon}_{m}+1})}}$ is the wavevector of the SPP along its direction of propagation [^3^]. For *λ_i_*=532 nm, the dielectric constant of Au is $\varepsilon_{m}$= 4.67 + *i*2.42,[^4^] and the estimated propagation length is about 1 µm.


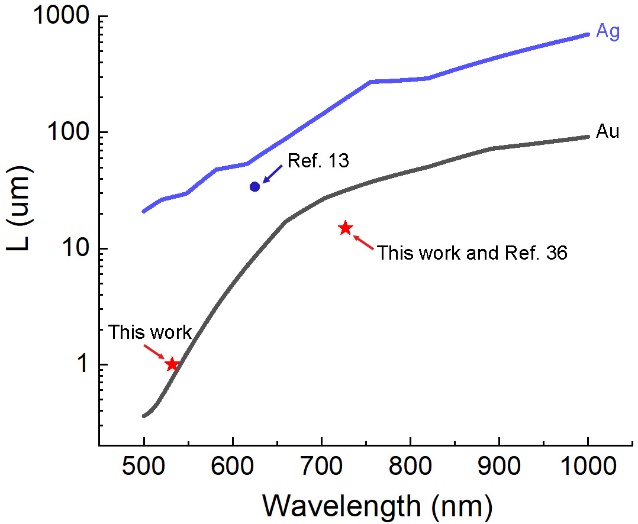


**Supplementary Figure 20.** Calculated SPP propagation lengths for Ag and Au using optical constants from optical constants from Johnson and Christy [^4^]. Measured values in this work, and Ref [13] and Ref [36] from the main text are shown. Ref [13] from the main text is listed below as Ref [^5^]. Ref [36] from the main text is listed here as Ref [^6^].

We performed 3D COMSOL simulations to obtain a qualitative picture for how modeled SPP decay occurs for a system analogous to the OPEN film structure. Supplementary Figure 19 shows results from simulations for a 50 nm thick Au film on 100 nm SiO2/Si, both with and without pores (model length = 15.5 μm, model width = 1.1 μm). The cross-sectional power dependence from these simulations is shown in the bottom plot for both the 532 nm and 727 nm excitation. In this plot we show a cross-sectional slice adjacent to the pores (dotted white line), where the decay is mostly monotonically decreasing.

The main conclusions from these simulations are: (i) for 727 nm excitation, the SPP decay length decreases by approximately 55% (from γ=20 μm to γ=9 μm) between ‘no pores’ and ‘pores’ and (ii) for 532 nm excitation the decay length is approximately the same between ‘no pores’ and ‘pores’ (γ=0.65 μm). This simulation result has a very similar trend to our experimental observations, where we measure an approximate 53% decrease in the decay length for γ=727 nm excitation from Ohmic loss alone (γ=32 μm) versus our measured OPEN-Au decay length (γ=15μm) and we find no decrease in the 532 nm decay length from Ohmic loss alone.


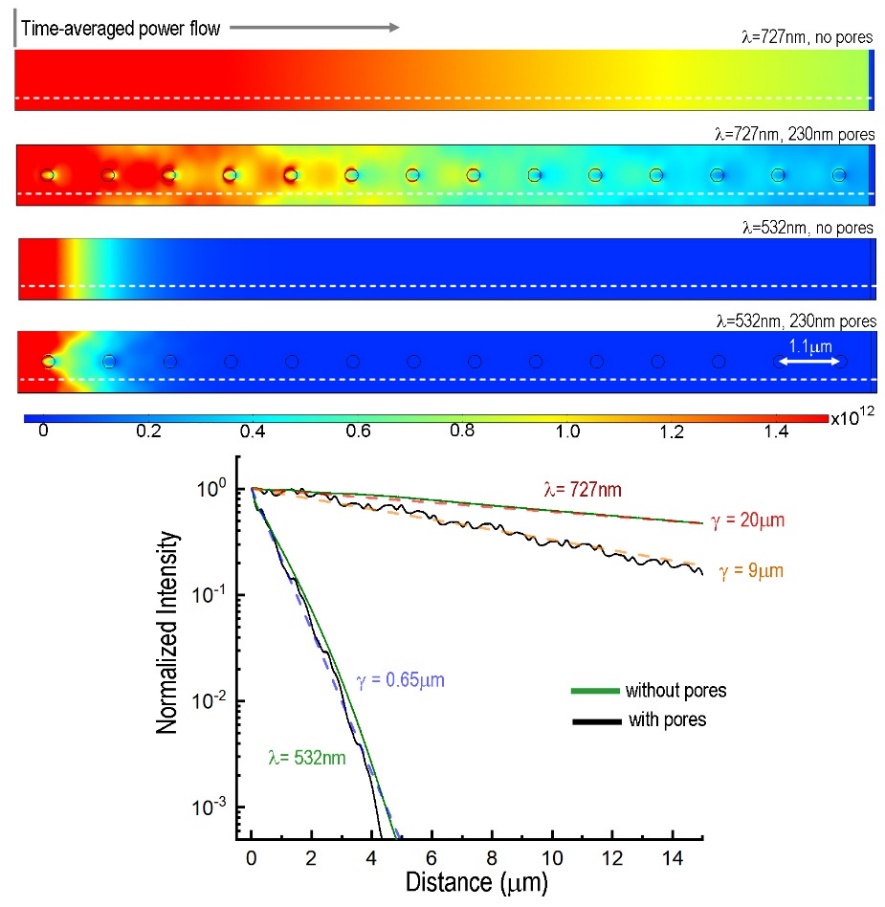


**Supplementary Figure 21.** 3D COMSOL simulation of a 50nm thick Au film on 100nm SiO_2_/Si with and without a line of pores (diameter=230nm, spacing=1.1μm; model length = 15μm, model width = 1.1μm). The power decay of SPP waves at different wavelengths is shown in the top three images. The SPP is launched from the left side of the image and propagates to the right side of the image. The bottom plot shows the normalized power cross-section (taken off center at 350nm from the edge; dotted white line) for 532nm and 727nm excitations, both with and without pores. We added an exponential decay (dashed lines) to show the approximate decay length (γ) for each model.

Supplementary Figure 20 shows an example of a high-resolution AFM image of another ‘sandwich’ Au/WSe_2_/Au sample in which pores form in both the top and bottom Au layer. We observe bright PL when pores in the top layer align with pores in the bottom layer, leaving either free-standing WSe2 or WSe_2_ touching the bottom SiO_2_ layer.


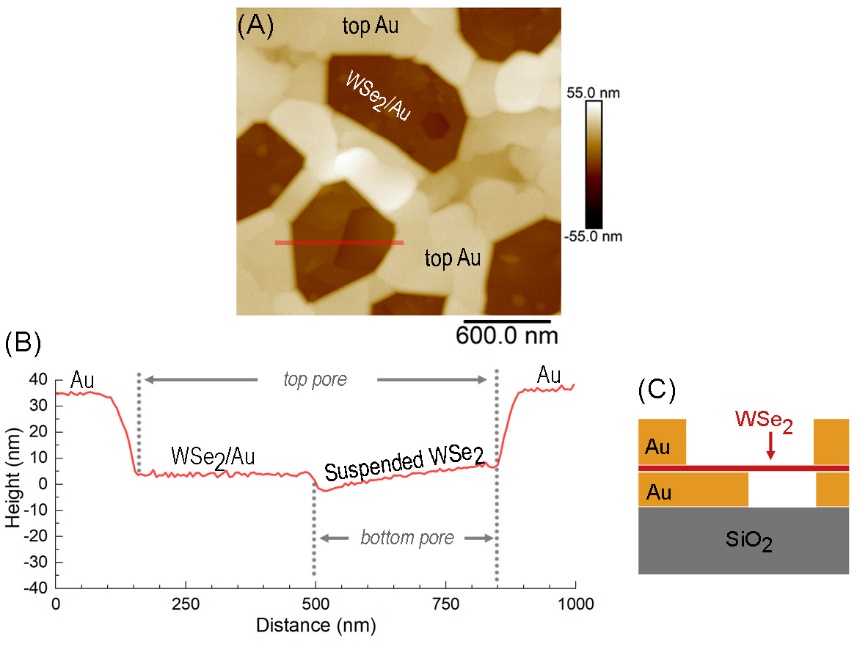


**Supplementary Figure 22:** (A) AFM height image of a ‘sandwich’ Au/WSe_2_/Au sample. (B) Cross-section profile from the red line in (A). (C) Schematic showing the location of the different layers in the structure.


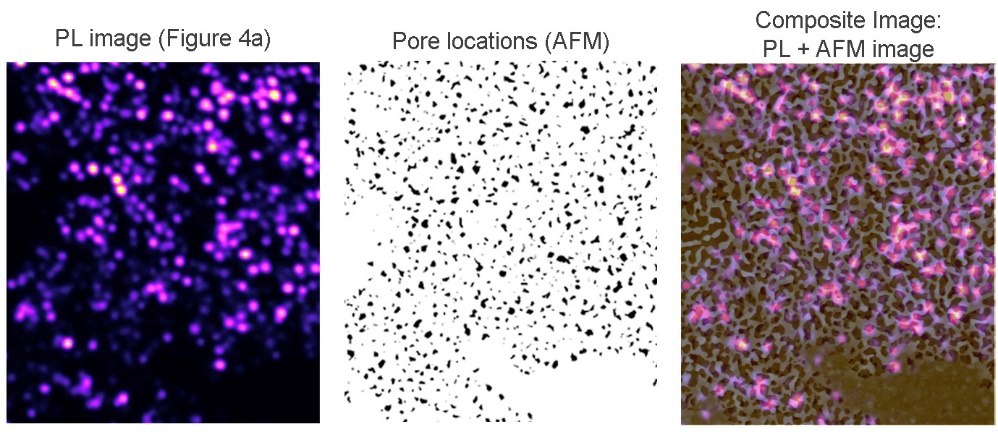


**Supplementary Figure 23:** Comparison of PL (integrated intensity) from a region in Figure 4a (left image) with the identified pores from AFM analysis (middle image). (Right image) Composite image with semi-transparent AFM height image (from Supplementary Figure 12A) overlaid with the PL map. The absence of PL/pores in the bottom right of the image is due to a hole in the WSe_2_ layer. Due to the absence of well-defined fiduciary marks, the image alignment is within one to two microns.


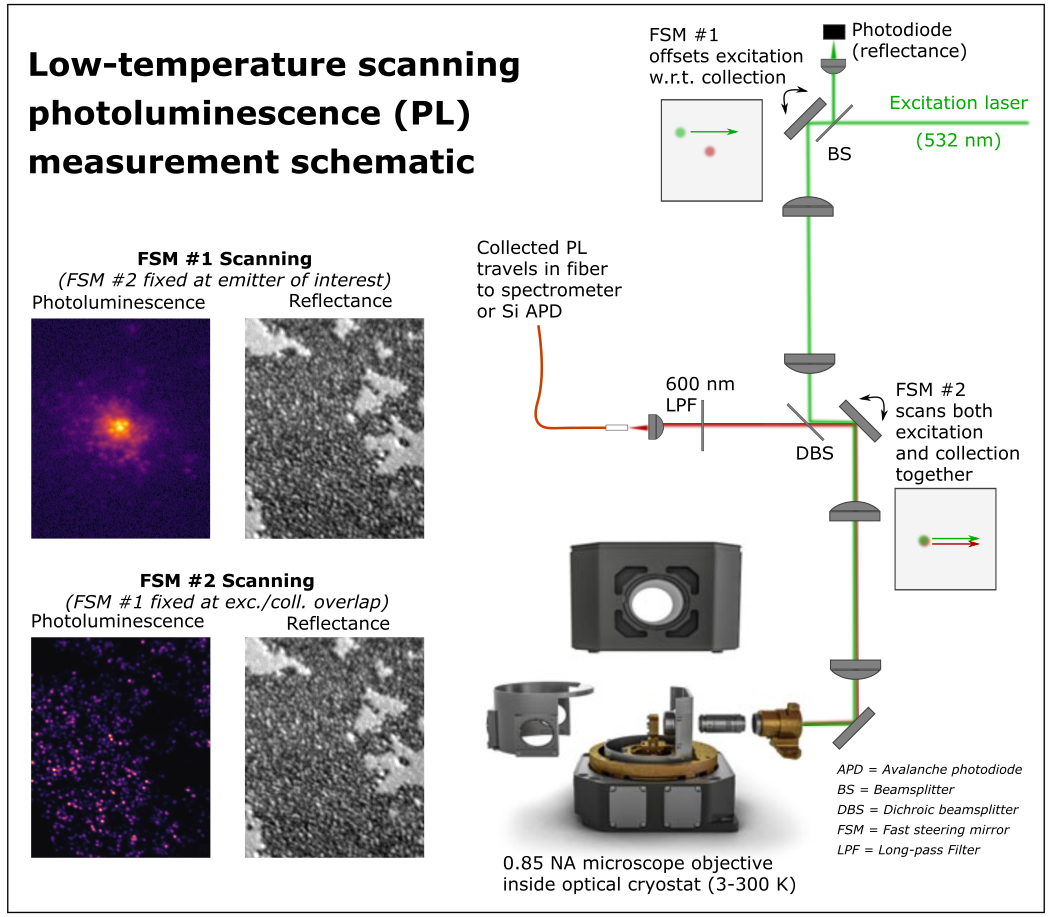


**Supplementary Figure 24.** Schematic showing routing of laser and sample mount for low-temperature measurements

**Supplementary References**

1 Flynn, R. A. *et al.* Transmission efficiency of surface plasmon polaritons across gaps in gold waveguides. *Applied Physics Letters* **96**, 111101 (2010).

2 Zhou, Y. *et al.* Probing dark excitons in atomically thin semiconductors via near-field coupling to surface plasmon polaritons. *Nature Nanotechnology* **12**, 856, (2017).

3 Maier, S. *Plasmonics: Fundamentals and Applications*. (Springer, 2007).

4 Johnson, P. B. & Christy, R. W. Optical Constants of the Noble Metals. *Physical Review B* **6**, 4370-4379, (1972).

5 Shi, J. *et al.* Cascaded exciton energy transfer in a monolayer semiconductor lateral heterostructure assisted by surface plasmon polariton. *Nature Communications* **8**, 35 (2017).

6 Kolomenski, A., Kolomenskii, A., Noel, J., Peng, S. & Schuessler, H. Propagation length of surface plasmons in a metal film with roughness. *Appl. Opt.* **48**, 5683-5691 (2009).
